# Transmesenteric Herniation through Congenital Mesenteric Defect leading to Bowel Gangrene

**Published:** 2015-05-01

**Authors:** Amna Bhatti, Amnah Azim, Samreen Khushbakht, Nasir Saleem Saddal

**Affiliations:** Department of Pediatric Surgery, National Institute of Child Health, Karachi, Pakistan

**Dear Sir,**

Transmesenteric herniation of bowel through a congenital defect in the mesentery of bowel is a rare surgical entity which may lead to bowel obstruction, ischemia, and gangrene.[1,2] The defect is congenital but patients may present at any age. We report a case of gangrenous bowel secondary to transmesenteric herniation.

A 5-year-old male presented with severe abdominal pain and non-bilious vomiting for 2 days along with abdominal distension for the last 24 hours. The patient was seen by a general practitioner who prescribed antipyretic and analgesics following which his pain settled for 10–12 hours. However, mother noticed abdominal distension on the next day. He did not pass stool for 12 hours. Despite abdominal distension the pain did not recur. On arrival in ER, the patient was lethargic and dehydrated with a respiratory rate of 42/min, pulse of 130 beats /min, blood pressure of 70/40mmHg and temperature of 100o F. Abdomen was grossly distended but not tender. On digital rectal examination rectum was empty. Nasogastric tube instantly drained 250 ml of bilious fluid. X-ray abdomen showed few dilated bowel gas shadows.

Following resuscitation patient was operated. On exploration, about 250ml of hemorrhagic fluid drained and small bowel loops delivered. A defect was seen within the mesentery of proximal ileum through which distal ileum was herniated and found gangrenous (Fig. 1). After widening the mesenteric defect, the herniated gut was delivered out (Fig. 2). About 25 cm of distal ileum was gangrenous. Resection of gangrenous part was done and ileoileal anastomosis made along with proximal diversion ileostomy. Postoperative recovery was uneventful.

**Figure:**
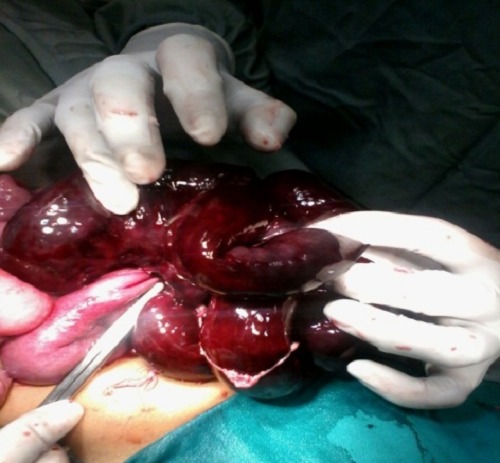
Figure 1: Gangrenous gut.

**Figure:**
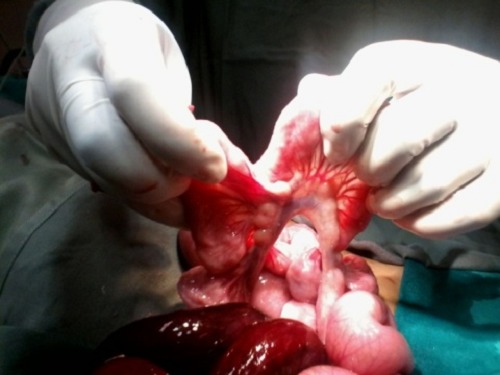
Figure 2: Mesenteric defect.

Internal hernia is a rare entity. It is a cause of small bowel obstruction in 0.5-5.8% of cases.[1,2] Congenital transmesenteric hernias constitute 5-10% of the internal hernias.[2] Multiple etiologies have been hypothesized as a cause of mesenteric defect including regression of dorsal mesentery, enlargement of hypovascular area, rapid lengthening of part of mesentery, compression of small bowel mesentery by transverse colon during physiological herniation etc.[2] Most of the mesenteric defects lie near the ileocecal mesentery while defects in jejunal or ileal mesentery are rare.[3] In our case, the defect was in the mesentery of proximal ileum.

The diagnosis of transmesenteric hernia presenting with intestinal obstruction requires high degree of suspicion and definitive diagnosis is made at laparotomy.[5,6] Surgical procedure is tailored according to the exploratory findings as well as patient’s condition. Gangrenous gut requires resection of the bowel as we did in our case.

## Footnotes

**Source of Support:** Nil

**Conflict of Interest:** None declared

